# Outcome measurement in perianal Crohn’s disease

**DOI:** 10.1007/s10151-026-03401-w

**Published:** 2026-08-11

**Authors:** Shivani Joshi, Ailsa Hart, Phil Tozer

**Affiliations:** 1 Department of Surgery and Cancer, Imperial College London, London, UK; 2 Robin Phillips’ Fistula Research Unit, St Mark’s Hospital and Academic Institute, London, UK

**Keywords:** Perianal disease, Crohn’s disease, Diagnosis, Outcome assessment, Patient-reported outcome measures, Quality of life

## Abstract

Perianal Crohn’s disease (pCD) is a distinct and debilitating phenotype of Crohn’s disease and an independent predictor of long-term adverse outcomes. Traditionally, outcome measurement has focused on fistula healing to assess treatment success; however, as we recognise distinct classes of patients and shift towards shared decision making, healthcare professionals are increasingly acknowledging the importance of assessing more nuanced outcomes such as downstaging, quality of life, sex and intimacy and the impact on family, education or work. Current challenges in outcome measurement include substantial heterogeneity in definitions, measurement tools and time points used. This review will cover the current landscape, reporting on historical measurement instruments, such as the fistula drainage assessment (FDA), perianal disease activity index (PDAI) and the collective move towards patient-centred and patient-developed tools, such as the Crohn’s anal fistula quality of life scale (CAF-QoL). It will also assess the value of core outcome measurement sets, which provide a uniform but flexible framework to standardise outcome reporting across studies and support comparison of interventions in clinical practice. Importantly, the incorporation of patient perspectives within their methodology ensures that their recommendations reflect outcomes that truly matter to those with pCD. The contemporary need is for novel clinical and radiological tools to monitor perianal disease activity that are predictive, preventative and provide treatment thresholds, so that patients can live well with their fistulising disease, if it cannot be eradicated. There will no doubt also be a role for digital applications and artificial intelligence in enhancing outcome measurement further for perianal Crohn’s disease.

## Introduction

Perianal Crohn’s disease (pCD) represents a complex, disabling manifestation of Crohn’s disease, associated with high relapse rates, substantial impairment in quality of life [1, 2] and long-term morbidity and functional limitation [3]. Outcome measurement in pCD must therefore reflect this breadth of impact, enabling accurate evaluation of the therapeutic efficacy of an intervention.

### Outcome measurement within the TOpClass classification

The Treatment Optimisation and Classification of perianal Crohn’s disease (TOpClass) is a new framework that was developed via international, multi-disciplinary consensus [4]. It has subsequently undergone retrospective functional validation in a real-world multi-centre cohort [5] and provides a granular, patient goal-directed approach to pCD management. As a relatively new framework, the role of TOpClass will continue to evolve as further evidence emerges. In contrast to the American Gastroenterological Association (AGA) classification, which broadly categorises fistulas as simple or complex, TOpClass recognises distinct clinical phenotypes which have differing treatment objectives and trajectories.

The TOpClass classification [4] identifies different classes of pCD on the basis of cross-sectional phenotypes. Over the course of disease, different classes require different treatment strategies and are characterised by different treatment goals. These goals may shift as understanding of their illness evolves, as circumstances change or as treatment is proactively driven to move from one class to another. As a result, outcome measurement must adapt to reflect the changing priorities relevant to each stage of the disease.

Traditionally, fistula closure, relevant for class 2a but rarely any other, has been the predominant outcome measure. However, this outcome is outdated and inadequately reflects the goals of many patients moving through the classification. It has limited value to both clinicians and those living with the disease, particularly outside of class 2a but even within it, in the absence of additional outcomes. For example, a patient in class 3 undergoing proctectomy has fundamentally different goals from one undergoing Ligation of the Inter-sphincteric Fistula Tract (LIFT) in class 2a. Similarly, a class 2b patient might wish to avoid a stoma, improve quality of life and reduce the impact of disease on work and social life, downstage to enable future repair or any combination of these goals. Outcome measurement needs to be flexible enough to identify success however it appears, yet consistent enough to permit comparison across treatments and study settings.

Other factors may influence outcome measurement; for example, patient goals may also drift over time, even if they remain within the same class. To begin with, many patients choose fistula eradication without risk. After years of failed attempts at repair and repeated interventions to control or downstage disease, some patients may instead choose a pragmatic truce with their fistula to prioritise other aspects of life, even though alternative options remain feasible. In this context, a well-placed seton with effective medical suppression of symptoms may enable these milestones more reliably than a well-meaning but unsuccessful advancement flap.

### Limitations of existing instruments

Current assessment tools remain limited in this regard. The perianal disease activity index [6] (PDAI, discussed in detail below) remains the primary measure of perianal disease activity, but we now need a scale which not only describes changes in perineal inflammation in response to treatment, but which also identifies the point at which a low probability of successful repair becomes a higher one, Similarly, magnetic resonance imaging (MRI) activity indices, also discussed below, demonstrate relatively limited responsiveness to change in any of the scores created. The newer scores (MAGNIFI-CD [7] and the TOpClass MRI definition of healing [8]) reliably detect a fully healed fistula, but what we need is an index which identifies improvement short of healing, predicts future complete radiological remission or identifies the threshold at which a higher chance of durable closure by surgical repair becomes likely.

In the context of a classification through which patients move, driven both by their disease progression and proactive treatment, and shaped by evolving personal goals, outcome measurement can no longer be restricted to the final treatment end point or outcome. Instead, it should capture clinically meaningful changes throughout the disease course, including key transitions between classes, achievement of intermediate treatment goals and maintenance of disease control.

As we review current outcomes and measurement instruments that are used, those which are fit for purpose and those requiring refinement, we suggest that their utility must expand to include nuanced determination of risk, prediction and patient-centred outcomes in a modern landscape of pCD management.

## Current landscape of outcome measurement in perianal Crohn’s disease

There has been a rapid evolution of treatment approaches for pCD in recent decades [9]. However, the modes for outcome measurement have not achieved comparable progress [10]. Substantial heterogeneity persists in defining key outcomes (fistula healing, recurrence, response) [11], the outcomes that are measured and measurement instruments employed [10], limiting data synthesis and the comparability of interventions, although the recent core outcome and core outcome measurement sets (CAF-COS [11] and CAF-COMS [10]) provide a framework for consistency.

## Clinician-reported outcomes

Historically, outcome measurement in pCD has been focused on trying to define healing of fistulae. Whilst this is often the initial patient goal, at many stages of the patient’s journey, fistula healing is a distant or even impossible aim. In addition, the absence of a cheap, easy, quick investigation to demonstrate healing in a robust way meant reliance on clinical indicators of fistula healing. Unfortunately, the only aspect of the fistula easily assessable in the clinic is the external opening and any discharge from it. Although the fistula drainage assessment (FDA) was developed to try to standardise this [12, 13], it fails to understand the tissues beneath the skin and whether deep tissue healing has occurred. Also crucially, the external opening of a fistula may close intermittently. This is the reason for cyclical abscess formation and the benefit of setons but also represents an opportunity for misdiagnosis of successful fistula healing if the assessment is simply made on the wrong day. The introduction of separate assessment visits attempts to reduce this risk but still represents a snapshot rather than a longitudinal assessment.

The perianal disease activity index (PDAI) [6] goes further, assessing the surrounding tissues and symptoms driven by perineal inflammation – the level of discharge, pain and physical and sexual restriction [14]. It remains very limited in its ability to assess fistula healing but also struggles to do more than describe the nature and symptomatic impact of the perineal inflammation. These tools lack rigorous methodology and, more significantly, have limited patient involvement in their development [15]. Better outcome measurement instruments are needed to assess fistula healing and in particular perineal inflammation, but also a wider range of targets which allow us to assess improvement throughout the patient’s journey, regardless of their class.

### Assessment of perineal inflammation

Nevertheless, assessment of perineal inflammation remains valuable. Looking forward and in light of the TOpClass classification, perineal inflammation determines the patient’s class through its presence or absence, dictates whether fistula repair is appropriate and could guide medical treatment in the same way as luminal markers of persistent inflammation despite treatment do. An objective measure of this inflammation, which is descriptive but also discriminative, in terms of suitability for repair and response to medical treatment is the goal of a new PDAI.

Both tools are used in clinical and research settings, with the PDAI inevitably performing slightly better than the deliberately narrow FDA in capturing a broader spectrum of symptom burden. However, both lack the ability to capture the wider scope of functional and psycho-social burden of disease [10]. Importantly, the PDAI is limited by its rigid structure that fails to detect mobility between fistula classes, e.g. downstaging from class 2b to 2a [4], and the need for treatment escalation on disease worsening. Other clinician-reported indices, such as the Allan Index [16], Pikarsky Index [17] and perianal disease severity score [18] were also evaluated as part of the development of a core outcome measurement set for fistulising perianal Crohn’s disease (CAF-COMS) and deemed limited in their clinical utility and psychometric validation [15].

### Downstaging

Downstaging is currently not recognised or measured by existing instruments as a distinct outcome in its own right. Biological downstaging describes the reduction in inflammation, which allows a fistula to become suitable for an attempt at repair and is distinct to anatomical downstaging, which reflects a reduction in fistula complexity to facilitate repair. Anatomical downstaging is achieved through a combination of optimised medical treatment and targeted surgical rationalisation and assessed before and after by MRI scans. Biological downstaging is achieved through medical treatment and sometimes defunctioning ostomy formation and has no validated measurement or monitoring modality. This deficiency hampers assessment of a meaningful and necessary outcome that should precede and predict subsequent anatomical repair.

### The need for a novel disease activity index

In the context of CAF-COMS, the PDAI was chosen as the ‘least worst’ of the existing instruments in measuring perianal disease activity. However, a novel instrument is needed to quantify inflammatory burden longitudinally and identify patients achieving clinically meaningful biological downstaging. This means not just those with active inflammation and those without, but also the more difficult task of identifying those whose perineum is sufficiently calm to facilitate an attempt at fistula repair – a theorised but not yet described group, who need to be identified to increase the rate of successful fistula repair. A biological marker of this may ultimately supersede a phenotypic description, but in its absence, an objective clinical assessment is required.

This new tool, or a modification of an existing tool, would need to for example evaluate the impact on quality of life, provide treatment thresholds and detect disease downstaging in line with the TOpClass classification [4]. Any new tool should be high-quality, predictive and dynamic and must track change short of fistula healing, escalating when a change in treatment may be required. As biological augmentation of structural repair operations advances, for example using autologous fat injections, identifying which patients will benefit from these adjuncts might also relate to perineal inflammation and assessing this clinically (in the absence of a biological marker) would also benefit from a robust assessment index.

This is particularly important given that radiological healing often lags behind clinical healing and symptom resolution by several months despite external opening closure. Clinical closure in the absence of radiological evidence of fistula healing overestimates the extent of durable healing [19] and is of limited value when used alone to define treatment success [20]. The PISA II study demonstrated that radiological improvement or healing correlates positively with durable fistula closure and improvement in quality of life [21, 22]; however, clinicians and researchers are increasingly recognising that the reduction of other symptoms such as pain and discharge and improvements in quality of life regardless of fistula healing are far more impactful on a patient’s daily life and must be reflected in the measurement instruments that are employed, both within and especially outside of class 2a.

### Other luminal/holistic scores

Despite their limited value in assessing perianal disease activity, other luminal/holistic scores such as the Crohn’s disease activity index (CDAI)[23] and Harvey−Brawshaw Index (HBI)[24] are frequently used instead of perianal disease activity tools. Whilst valuable in assessing luminal disease activity, they have limited clinical utility in identifying changes in perianal discharge, pain or fistula healing [13, 25].

Clinician-reported outcome measures are important in the assessment of perianal disease activity and longitudinal monitoring and will remain so. However, it is vital that they are used with caution within research studies when used independently, unless modified significantly. The high levels of subjectivity, inter-observer variability, partial construct validity and focus on physical symptoms alone [26] mean that without the use of an additional patient-reported outcome measure (PROM), the study may not provide an effective evaluation of the true benefit of an intervention, using outcomes that matter to patients. Acknowledging these flaws in clinical outcome measurement, and their limited applicability outside class 2a, catalyses a necessary shift towards more integrated frameworks that combine clinician and patient-reported outcomes, in parallel with radiology to accurately capture clinical improvement, downstaging and resolution of disease.

## Patient-reported outcomes and quality of life

Outcome assessment in pCD has conventionally captured few elements of the patient’s lived experience, especially on an intimate and functional level. Many patients are diagnosed during their formative years, at the start of their personal, sexual and professional lives [27, 28], when this impact may be most destructive. The daily impact of pain, discharge, smell, fatigue and fear of incontinence can be profound, leading to embarrassment, anxiety and social withdrawal [1, 29]. These psycho-social burdens are not adequately reflected in traditional clinician-reported measures that focus predominantly on disease activity and fistula closure [26]. Thus, there is a growing trend towards developing patient-reported outcome measures (PROMs) to capture these challenges [30].

The Crohn’s anal fistula quality of life (CAF-QoL) scale is the most widely used disease-specific PROM developed for pCD [30]. Its development followed a rigorous, multi-phase process guided by the COSMIN (COnsensus-based Standards for the selection of health Measurement INstruments) framework [31] with substantial patient input at every stage. CAF-QoL has demonstrated excellent psychometric properties, including high internal consistency, reliability and sensitivity to change [30]. It is notable in its inclusion of emotional wellbeing, fatigue and sexual function, key components of quality of life that are often neglected [30]. The robust methodology and patient centrality have established it as a leading instrument for patient-reported outcome assessment in pCD [30].

Other quality of life (QoL) instruments employed within pCD research include the Inflammatory Bowel Disease Questionnaire (IBDQ) [32] and its short-form version (SIBDQ)[33]. These capture physical, emotional and social wellbeing, but are generalised to inflammatory bowel disease (IBD) populations [32, 33] and have not been specifically validated in patients with pCD [32]. As a result, they do not address symptoms such as persistent perianal discharge, pain or the impact on sex and intimacy, leading to potential underestimation of the disease burden in this patient cohort. Similarly, the EQ-5D is another generic tool widely used in perianal Crohn’s studies, however it only includes five broad domains, which lack specificity or sensitivity to the subtle complexities related to perianal fistulising disease [34].

### Areas for improvement

There are several areas for improvement if we are to truly capture patient-reported outcomes (PROs) within pCD research. Currently, the choice of PROM, the time points at which measurements are taken and the level of weighting given to patient-reported outcomes within studies remain inconsistent [10, 11]. The persistent use of generic PROMs rather than those designed specifically for pCD is likely to underestimate the true impact of an intervention [11]. Finally, whilst the development of PROMs is a significant step in acknowledging the hidden impacts of living with the disease, the often deep and complex emotions and psycho-social impact faced by individuals with pCD may not be fully quantifiable in any objective measure. However, although nuanced, capturing these PROs is an essential step towards translating complex lived experiences into measurable outputs to meaningfully inform research and evidence-based decision-making.

The inclusion of quality of life in CAF-COS has cemented it as a key outcome that should be included in all interventional pCD studies [11]. This inclusion should facilitate the integration of quality of life as a routinely measured outcome within studies, and where appropriate, as a primary outcome.

### Sex and intimacy

Perianal Crohn’s disease can have a profound impact on sex, intimacy and body confidence; however, these aspects are rarely discussed in clinical consultations or within outcome measurement [35]. Preliminary findings from a systematic review suggest that existing studies often use generic tools, such as the Female Sexual Function Index (FSFI) or International Index of Erectile Function (IIEF), which fail to capture the disease-specific or less physical effects such as altered body image, loss of sexual confidence, spontaneity and relational impacts [36].

The development of a sexual function patient-reported outcome measure (Sex-PROM) is underway to measure the impact of benign pelvic and perineal colorectal surgery on sex and intimacy. This is being developed with representation across a wide range of ages, genders, ethnicities and sexual orientations. Preliminary qualitative findings suggest that fistula surgery can affect sexual activity, sexual practices and positions (including but not exclusively ano-receptive intercourse), cause increased discomfort, change in sensation, psychological distress and loss of self-esteem. Preliminary data also suggest pCD-specific concerns relating to unpredictable or persistent discharge, smell and associated stigma, intensifying anxiety around sexual activity often leading to avoidance [37].

## The role of core outcome measurement sets in pCD research

The core outcome measures in effectiveness trials (COMET) framework [38] has encouraged the development of core outcome measurement sets within healthcare research, facilitating standardised outcomes and outcome measurement instruments within all studies for a specific health condition [39].

The core outcome set for Crohn’s anal fistula (CAF-COS), developed by Sahnan et al. [11], represented a major milestone in harmonising outcome reporting in this complex field, and followed a rigorous, multi-disciplinary process involving a systematic review, qualitative patient interviews and an international Delphi consensus. This multi-disciplinary, global approach strengthened the validity and generalisability of the COS, which identified three overarching domains: clinician-reported outcomes, patient-reported outcomes and an optional radiological module [11].

The TOpClass consortium has developed a core outcome measurement set for Crohn’s anal fistulas (CAF-COMS) [10]. These preliminary findings have identified optimal measurement instruments and time points for each outcome in the COS. CAF-COMS also identified whether existing best measurement instruments were ideal, or whether a new instrument was required. Perianal disease activity, continence impairment and radiological disease activity were all outcomes for which there is no ideal measurement instrument and a new or modified tool is required.

**Fig. 1:**
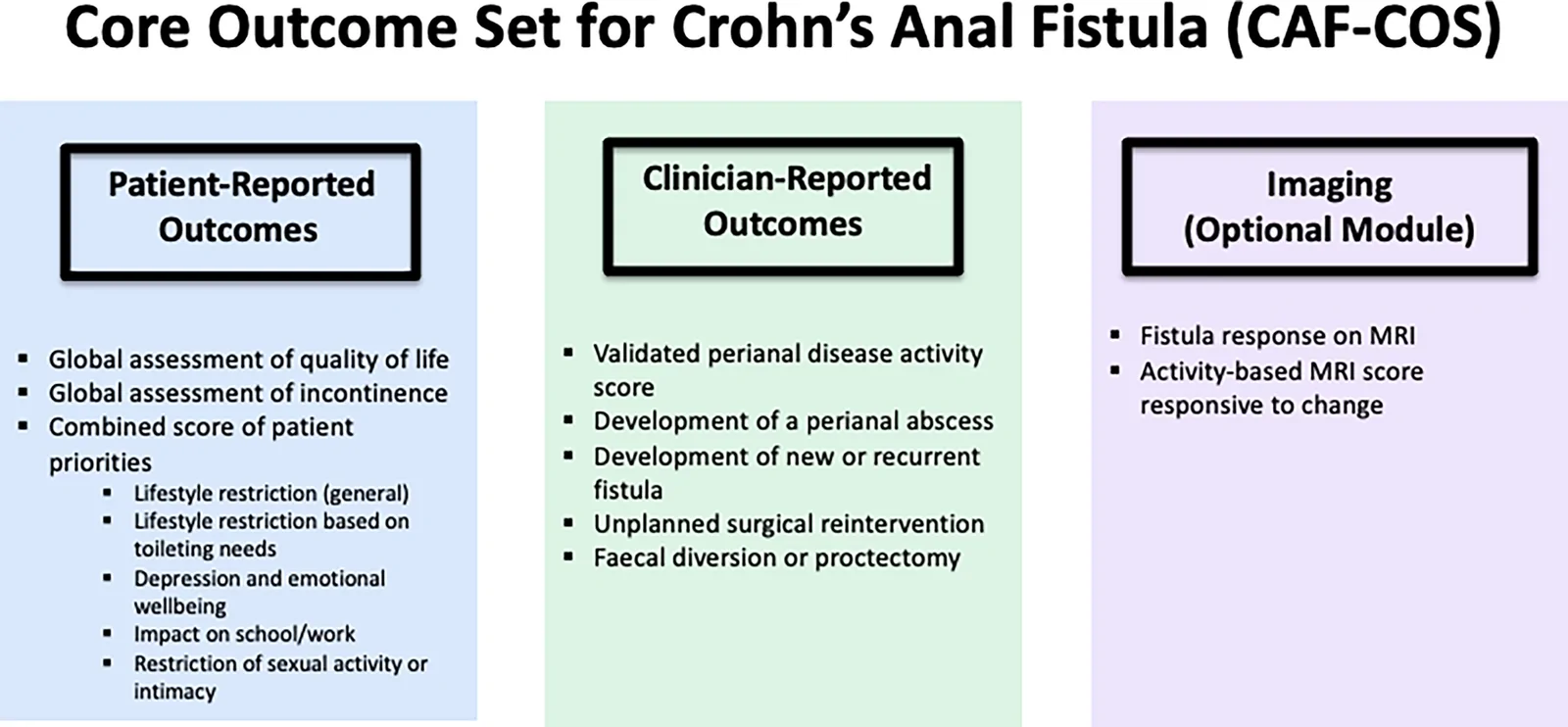
Core outcome set for Crohn’s anal fistula (CAF-COS), adapted from Sahnan et al. [11]

The adoption of core outcome sets and measurement sets within future clinical trials and real-world studies for perianal Crohn’s disease will enable uniform, comparable reporting. This coordination will enhance the quality, reproducibility and clinical relevance of studies, improving the translation of research findings into practice. It will facilitate robust meta-analysis and data synthesis, incorporating patient perspectives to shift towards a patient-centred description of success in perianal Crohn’s disease treatment [Fig. 1].

## Continence

For those living with pCD, altered continence, persistent leakage and secondary social avoidance despite achieving clinical and/or radiological healing after an intervention is common; thus, it was chosen as a key outcome in CAF-COS. [11].

### Limitations of existing continence scores

However, the evaluation of continence within fistula research remains problematic. Commonly used instruments, such as the Vaizey or Wexner incontinence scores, were originally developed for post-operative/idiopathic anal incontinence, not pCD [40, 41]. These tools often fail to distinguish between pre-intervention pad usage and fistula discharge, and post-intervention anal incontinence, producing misleading results. For example, a patient may report high incontinence scores pre-intervention owing to fistula drainage and then maintain similarly high scores post-intervention owing to the presence of actual incontinence in the presence of a healed fistula. As a result, the actual change in continence level after an intervention is concealed. This error is amplified in cohort-level analyses, which often aggregate scores across a cohort before surgery and compare the resulting number with a similar aggregate across the cohort afterwards, hiding the group of patients with worsened continence amongst those with little or no recorded change.

### Development of a fistula-specific incontinence score

The minimum reporting standards (MRS) for anal fistula research highlighted that generic incontinence scores are frequently employed inappropriately within Crohn’s-related fistula studies without specific adaptation [42].The standards advise that analysis is required at individual patient level to ensure a meaningful interpretation of results. The presence of these methodological challenges has prompted the TOpClass consortium to begin developing a fistula-specific continence measure that may accurately separate fistula drainage from genuine incontinence, whilst also encompassing broader holistic impacts, such as functional confidence and secondary social participation.

## Radiological disease activity measures

Magnetic resonance imaging (MRI) remains the gold standard for assessing perianal disease activity, providing a detailed evaluation of fistula tract morphology, particularly the presence of secondary extensions and deeper pockets of inflammation. It also allows for pre-operative mapping, treatment planning and longitudinal monitoring [43].

### Existing MRI-scoring systems

Several MRI-scoring systems exist to quantify perianal disease activity and fistula healing. These include the Van Assche score (VAS), modified Van Assche score (mVAS) and, more recently, the MAGNIFI-CD. These were found to show significant heterogeneity amongst component domains and definitions for ‘fistula healing’ [8].

The Van Assche score (VAS) is the most frequently utilised tool across fistula studies, incorporating the number of tracts, extent of disease, T2 hyperintensity and the presence of abscesses or rectal wall involvement. Despite providing a useful structural outline of fistula anatomy, it does not provide sufficient information on disease activity or responsiveness to change [14, 42]. In a move to improve its sensitivity, the modified Van Assche score (mVAS) was developed [45]. While more pragmatic, subsequent validation studies have shown only a modest improvement in responsiveness, with performance overall similar to the original score [46]. Further attempts to improve on this include the development of MAGNIFI-CD [7]. This score quantifies radiological disease activity via T2 hyperintensity, post-contrast enhancement and diffusion restriction. Validation studies have shown improved inter-rater reliability, although external validation and responsiveness remain limited [37], perhaps owing to the continued focus on structural features, some of which can only change when full healing occurs.

Overall, these scoring systems are limited by their focus on structural features, lack of standardisation between studies and limited predictive value [8]. A consensus definition for a radiologically healed Crohn’s anal fistula, outlined by absence of T2 hyperintensity or contrast enhancement on post-contrast T1 weighted images and a completely fibrotic fistula tract has been developed [8]. The TOpClass consensus definition does not require the use of contrast and is sensitive for identifying a healed fistula. However, no radiological activity score is sufficiently responsive to change short of full healing nor can it discriminate between fistulae on the basis of previous treatment or predict outcomes.

### Fistula volume as a novel marker of disease activity

Fistula volume has emerged as a novel, objective marker of disease activity that provides a dynamic, three-dimensional (3D) model capable of identifying change over time [47]. It has the potential to support treatment planning and facilitate communication of disease status and treatment response clearly with patients [47]. However, its current use is largely confined to expert centres and research settings. Wider implementation will require demonstration of acceptable reproducibility and feasibility across centres before adoption within routine clinical practice.

Volume and other radiomics are also being evaluated by various groups, and an automated or easy to use scoring scale which is discriminative and predictive as well as descriptive will meet the ideal requirements identified in the COS and COMS.

Existing radiological scores prioritise anatomical healing and struggle to capture reduced perineal inflammatory burden, i.e. biological downstaging. This is an intermediate, but clinically meaningful outcome which describes a move from class 2b to class 2a and opens an opportunity to undertake repair and achieve durable fistula healing. Future radiological work may quantify perineal inflammation (perhaps using an ultrasound technique), demonstrate responsiveness to therapy and predict the likelihood of treatment success, in complement with a clinical disease activity index.

### Future directions

The development of core outcome and measurement sets [11] has provided the foundation for consistent, reproducible, patient-centred and meta-analysable outcome measurement in pCD. Future research must align with these recommendations to enable meaningful comparison of therapeutic efficacy, with additional outcomes bespoke to specific research studies bringing valuable nuance. True success varies per individual and across the disease trajectory, extending beyond or away from fistula tract closure to restoration of functional confidence, psychological wellbeing and quality of life. Outcome assessment must remain adaptable to evolving disease course, treatment strategy and patient priorities, with increasing emphasis on class-specific outcomes assessed longitudinally rather than at fixed time points.

A natural progression in work on end points is the emergence of treat-to-target (T2T) strategies within inflammatory bowel disease, exemplified by the STRIDE [48, 49] and SPIRIT [50] initiatives for luminal disease. The TOpClass consortium, in collaboration with International Organization for the Study of IBD (IOIBD), is developing a T2T strategy for pCD, defining early-term, mid-term and late-term targets, delineated by fistula class [4]. This represents a shift towards personalised, proactive target-driven management for those with pCD, aimed not only at predicting predict trajectory but preventing progression and facilitating downstaging.

**Fig. 2:**
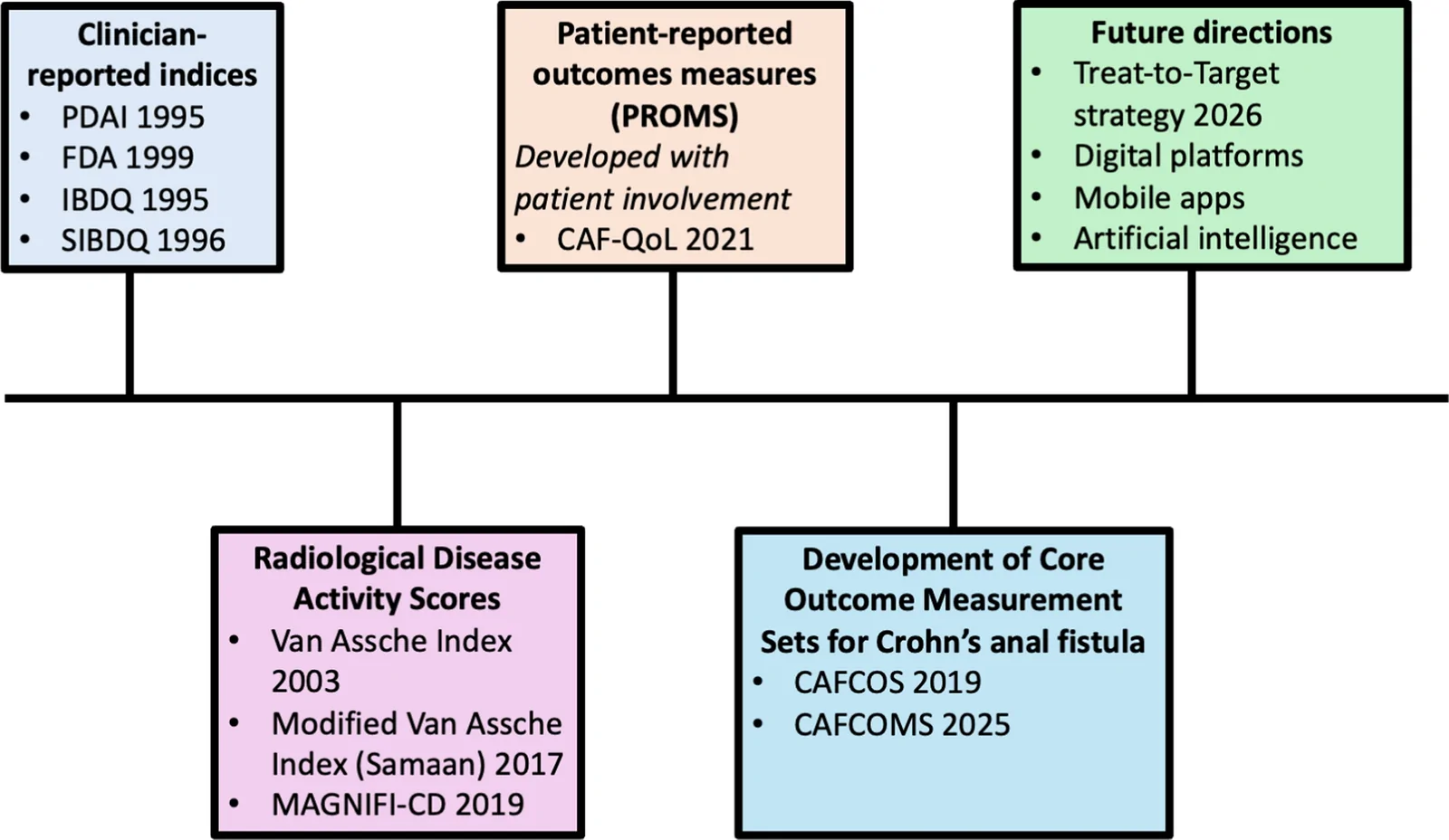
Evolution of outcome measurement in perianal Crohn’s disease

Digital health technologies and artificial intelligence (AI) offer further potential, when used with care. Mobile applications and longitudinal monitoring interfaces may allow the continual tracking of composite outcomes with minimal user burden [51–53]. These platforms could support earlier detection of relapse, identification of therapeutic response or downstaging, prompting opportunities for fistula repair [Fig. 2].

## Conclusions

Foundational work in outcome measurement has created a landscape for research which makes possible patient-centred, consistent and meaningful measurement of successful treatment of perianal Crohn’s fistula. Some existing outcome measurement instruments are imperfect and others unfit for purpose. The new treatment paradigm for perianal disease is personalised, proactive and multi-disciplinary and utilises combined medical, surgical and cell therapy interventions, all on the basis of a pragmatic, patient-centred classification.

The next phase of outcome measurement needs to include tools which are descriptive but also discriminative and predictive, to help identify when patients move from one class to another, to identify intervention opportunities for downstaging and prevention of disease progression and to help determine when patients reach a point of disease control and quality of life restoration.

These changes in the landscape recognise the lifelong nature of IBD and the variation in impact and treatment needs across the different classes of pCD. Longitudinal assessment, personalisation of both intervention and measurement of success, with consistent, reproducible assessments that guide treatment are a crucial part of the modern management of pCD.

## Data Availability

No datasets were generated or analysed during the current study.

## Declarations

### Patient and public involvement

No patients were involved in the development of this manuscript.
